# Key ingredients in *Verbena officinalis* and determination of their anti-atherosclerotic effect using a computer-aided drug design approach

**DOI:** 10.3389/fpls.2023.1154266

**Published:** 2023-04-03

**Authors:** Yuting Chen, Yuanyuan Gan, Jingxuan Yu, Xiao Ye, Wei Yu

**Affiliations:** ^1^ School of Pharmacy, Xianning Medical College, Hubei University of Science and Technology, Xianning, Hubei, China; ^2^ Clinical Medical College, Changsha Medical University, Changsha, Hunan, China; ^3^ Hubei Engineering Research Center of Traditional Chinese Medicine of South Hubei Province, Xianning, Hubei, China

**Keywords:** *Verbena officinalis* (VO), atherosclerosis (AS), network pharmacology approach, molecular docking, molecular dynamics simulation

## Abstract

Lipid metabolism disorders may considerably contribute to the formation and development of atherosclerosis (AS). Traditional Chinese medicine has received considerable attention in recent years owing to its ability to treat lipid metabolism disorders using multiple components and targets. *Verbena officinalis* (VO), a Chinese herbal medicine, exhibits anti-inflammatory, analgesic, immunomodulatory, and neuroprotective effects. Evidence suggests that VO regulates lipid metabolism; however, its role in AS remains unclear. In the present study, an integrated network pharmacology approach, molecular docking, and molecular dynamics simulation (MDS) were applied to examine the mechanism of VO against AS. Analysis revealed 209 potential targets for the 11 main ingredients in VO. Further, 2698 mechanistic targets for AS were identified, including 147 intersection targets between VO and AS. Quercetin, luteolin, and kaempferol were considered key ingredients for the treatment of AS based on a potential ingredient target–AS target network. GO analysis revealed that biological processes were primarily associated with responses to xenobiotic stimuli, cellular responses to lipids, and responses to hormones. Cell components were predominantly focused on the membrane microdomain, membrane raft, and caveola nucleus. Molecular functions were mainly focused on DNA-binding transcription factor binding, RNA polymerase II-specific DNA-binding transcription factor binding, and transcription factor binding. KEGG pathway enrichment analysis identified pathways in cancer, fluid shear stress, and atherosclerosis, with lipid and atherosclerosis being the most significantly enriched pathways. Molecular docking revealed that three key ingredients in VO (i.e., quercetin, luteolin, and kaempferol) strongly interacted with three potential targets (i.e., AKT1, IL-6, and TNF-α). Further, MDS revealed that quercetin had a stronger binding affinity for AKT1. These findings suggest that VO has beneficial effects on AS *via* these potential targets that are closely related to the lipid and atherosclerosis pathways. Our study utilized a new computer-aided drug design to identify key ingredients, potential targets, various biological processes, and multiple pathways associated with the clinical roles of VO in AS, which provides a comprehensive and systemic pharmacological explanation for the anti-atherosclerotic activity of VO.

## 1 Introduction

Atherosclerosis (AS) is a chronic inflammatory disease characterized by lipid deposition on vessel walls (Libby et al., 2011). Several risk factors are associated with its development, including hyperlipidemia, hypertension, chronic stress, glucose metabolism disorders, smoking, genetics, aging, and sex-related factors (Yao et al., 2019; Landowska et al., 2022). In modern society, the mortality and morbidity of AS are increasing; at present, it is a leading cause of death among adults (Dahlöf, 2010). The World Health Organization states that approximately 17.9 million people die from cardiovascular diseases every year, accounting for 32% of all deaths in both developed and developing countries (Fan and Watanabe, 2022). AS leads to endothelial dysfunction, the formation of a new endothelial layer, and the development of lipid deposits, foam cells, and plaques, which eventually rupture, resulting in the formation of thrombi and the narrowing of blood vessels. Further, it also causes ischemia and hypoxia in the tissues and organs involved, leading to symptoms such as chest tightness and pain and even sudden death sometimes (Grootaert et al., 2018). To date, the mechanism underlying AS remains unelucidated; however, studies suggest that it is associated with lipid metabolism disorders, inflammation, oxidative stress, autophagy disorder, and mitochondrial dysfunction (Zhang et al., 2021; Chen et al., 2022a). At present, three main types of statins are used in clinical settings: atorvastatin, resulvastatin, and lipocalin. Further, some fibrates, such as benzofibrate and fenofibrate, and aspirin are used as antithrombotic medications. However, statins are prone to muscle toxicity, including myositis, mild hypercreatine kinaseemia, and acute liver failure owing to rhabdomyolysis (Hilton-Jones, 2018). In addition, statins and fibrates can affect liver function, resulting in elevated alanine transferase and aspartate transferase levels, usually in the early stages, mostly because of the drug dose. This may lead to changes in hepatocyte membrane structure, mitochondrial dysfunction, apoptosis, and drug interactions. As a result, safe and effective medications are urgently warranted.

In traditional Chinese medicine, *Verbena officinalis* (VO) is a commonly used herb with abundant sources, a low price, and low toxicity. It was originally published in *Mingyi Bielu* (Records of Famous Physicians). VO is a perennial herb that grows worldwide in Europe, America, North and Central Africa, Asia, and Australia (Austin, 2004). It stimulates blood circulation, disperses blood stasis, clears heat, detoxifies the body, and suppresses coughing. High-performance liquid chromatographic analysis revealed that VO mainly comprises flavonoids, iridoid glycosides, triterpenoids, phenylpropanoids, and other components (El-Hela et al., 2010; Gibitz-Eisath et al., 2018). With bitter and cold properties, VO exerts its influence *via* the liver and spleen meridians. According to modern pharmacological research, the active ingredients in VO are lipid regulators and possess anti-inflammatory, analgesic, immunomodulatory, and neuroprotective properties. Owing to its ability to inhibit lipid metabolism (Steinberg, 2004; Konstantinov et al., 2007), some of its ingredients may be useful for treating AS (Tu et al., 2007; Xue et al., 2017); however, whether VO has an anti-atherosclerotic effect *via* multiple components and targets remains unclear.

**Figure 1:**
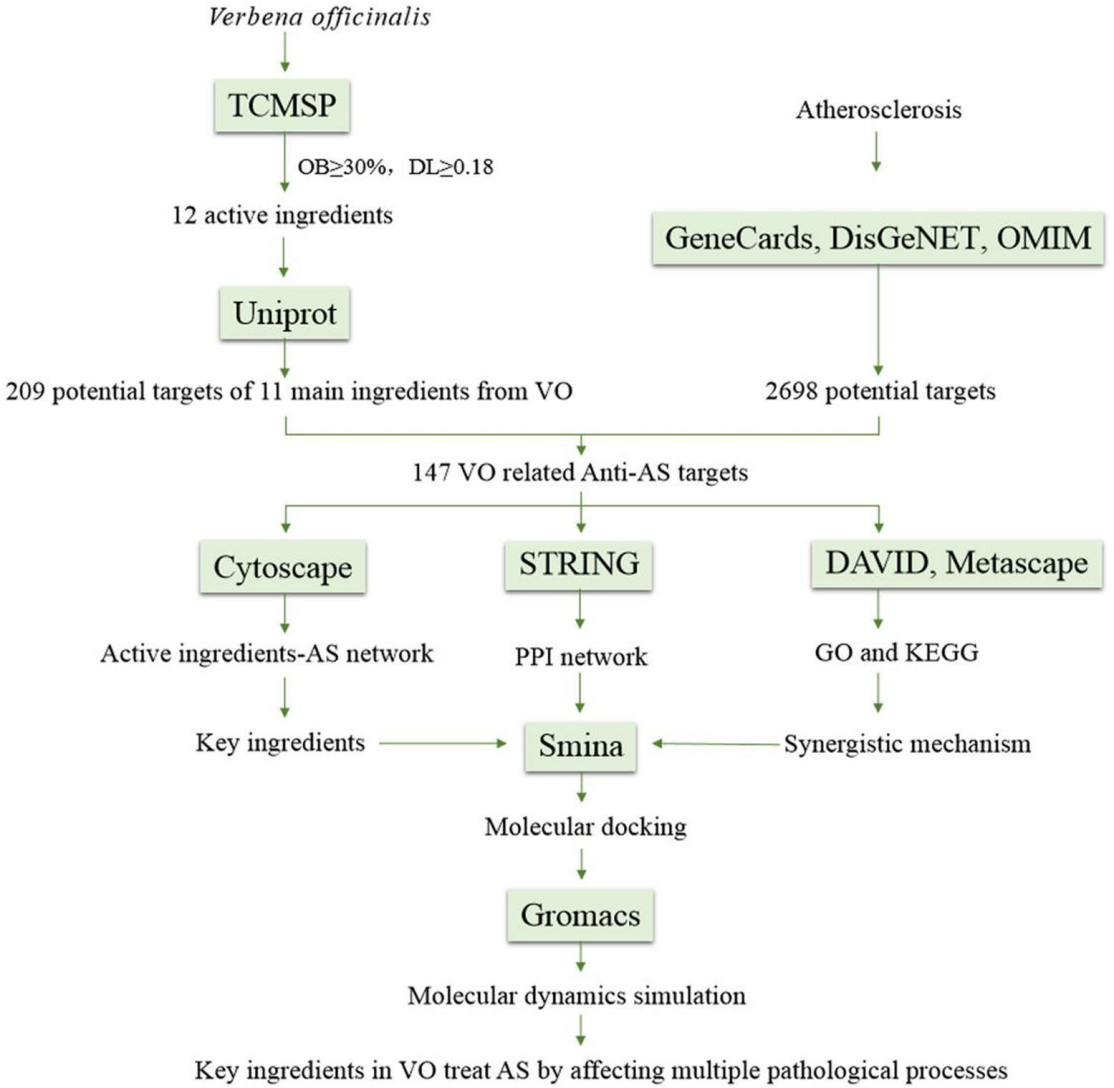
Flow chart showing the steps involved in investigating the pharmacological mechanism of VO against atherosclerosis.

A computer-aided drug design method combines network biology and multipharmacology, including molecular docking and molecular dynamics simulation (MDS), to develop an updated drug discovery method. In the present study, network pharmacology was used to examine the key ingredients in VO and its possible targets against AS. Further, molecular docking and MDS were used to explore the interaction between targets and active ingredients in VO. In addition to these techniques, we used a computerized target prediction system based on interactions among components in medicinal materials and targets and among genes and biological pathways to determine the relationship comprehensively and systematically between active ingredients in VO and their anti-atherosclerotic effects for the first time (**Figure 1**).

## 2 Materials and methods

### 2.1 Database websites and software

The databases and software used were as follows: Traditional Chinese Medicine Systems Pharmacology (TCMSP; http://tcmspw.com/tcmsp.php), ClassyFire (https://cfb.fiehnlab.ucdavis.edu/), GeneCards (http://www.genecards.org), DisGeNET (https://www.disgenet.org/), OMIM (https://www.omim.org/), PANTHER Classification System (http://pantherdb.org/), UniProt (https://www.uniprot.org), Venny2.1.0 (http://bioinfogp.cnb.csic.es/tools/venny), STRING (http://string-db.org/cgi/input.pl), DAVID (http://David.ncifcrf.gov), Bioinformatics (https://www.bioinformatics.com.cn/), Metascape (https://metascape.org/gp/index.html#/main/step1), PubChem (https://pubchem.ncbi.nlm.nih.gov/), Cytoscape 3.9.0 software, Smina software, Gromacs 2019.6, and Gmx_MMPBSA software.

### 2.2 Network pharmacology

#### 2.2.1 Collection of the active ingredients in VO and targets

Using the TCMSP database (Ru et al., 2014), the active ingredients in VO were identified according to the following basic criteria: oral bioavailability ≥30% and druglikeness ≥0.18 (Tao et al., 2012; Xu et al., 2012). PubChem was used to obtain the InChIKeys for each ingredient in VO. These InChIKeys were then entered into the ClassyFire database for classification (Djoumbou Feunang et al., 2016).

To obtain targets related to the main ingredients in VO, data were retrieved from the TCMSP database, the Uniprot database was searched for all verified human genes and their corresponding gene names, and the target genes of VO were annotated according to their database names to determine potential action targets for the active ingredients in VO (UniProt Consortium, T, 2018). Then, genes with duplicate values were removed (such as *NOS2*, *PTGS1*, *PTGS2*, and *DPP4*), followed by the construction and visualization of an interconnected network graph of the effective ingredients and targets using Cytoscape 3.9.0 software (Chai et al., 2022).

#### 2.2.2 Screening VO targets correlated with AS pathology

Data from the GeneCards (Safran et al., 2010), DisGeNET (Zhao et al., 2021), and OMIM (Amberger et al., 2015) disease databases were retrieved using the keyword “AS.” We selected disease targets with a corresponding median of ≥1.12 based on GeneCards data. Simultaneously, disease targets with a score of ≥0.02 in the DisGeNET database were screened. Finally, AS targets were obtained by combining the disease targets from the three databases and removing duplicates. The targets for VO and AS were imported into Venny 2.1.0 to prepare Venn diagrams, and VO targets correlated with AS pathology were obtained (An et al., 2022).

#### 2.2.3 Functional classification of potential VO targets

VO targets were functionally annotated and classified using the Panther classification system (Mi et al., 2019), in which the VO targets were classified using *Homo sapiens* as the only organism. Results were plotted using Origin 2017 software.

#### 2.2.4 Construction of the protein–protein interaction (PPI) network

The PPI network of the target proteins was constructed using the STRING11.5 database (Szklarczyk et al., 2016) and visualized using the Cytoscape 3.9.0 software (Shannon et al., 2003). A significant interaction score was determined as >0.4 only for the organism *H. sapiens*. Fifteen core targets were chosen based on their degree values, which were determined using NetworkAnalysis (a Cytoscape plugin).

#### 2.2.5 Gene ontology (GO) and Kyoto Encyclopedia of Genes and Genomes (KEGG) pathway enrichment analyses

A GO analysis of biological functions was performed using the DAVID database (Huang et al., 2009; Chen et al., 2017), which comprises biological processes (BP), cell components (CC), and molecular functions (MF). Terms were considered statistically significant when the p-value was<0.05. The top 10 terms were visually represented using an online drawing program called Bioinformatics (Xia et al., 2022). Enrichment bar graphs were created for KEGG pathways for the species *H. sapiens* using the Metascape database (Zhou et al., 2019b).

### 2.3 Molecular docking

To validate the binding of the active ingredients in VO to the core targets, the PubChem database (Kim et al., 2020) was used to determine the three-dimensional (3D) structures of the active ingredients, and AlphaFold2 was used to obtain the protein structure files of the targets (Jumper et al., 2021). Molecular docking calculations were performed using the molecular docking software Smina (Masters et al., 2020). The size of the docking box was 126, and the docking approximation was 20, with 20 conformations generated each time. When a ligand binds to its receptor, hydrogen bonding and hydrophobic interactions are formed; hydrogen bonding plays an essential role in maintaining ligand–receptor binding stability. When a ligand binds with a receptor, it changes the structure and chemical properties of the receptor to obtain the ligand–receptor complex system with good stability.

### 2.4 MDS

The software Gromacs 2019.6 (Van Der Spoel et al., 2005), Amber14sb for proteins, Generation Amber Force Field for macromolecules, and TIP3P water model for water were used in addition to a sodium ion balance system to provide the complex system with a balanced electrolyte solution. The Verlet and Cg algorithms were used for elastic simulation. The particle mesh Ewald method was used to analyze electrostatic interactions, and the steepest descent method (5,000 steps) was chosen to minimize energy. Van der Waals (VDWAALS) radius and Coulomb force cutoff distances were both set at 1.4 nm. After balancing the regular and isothermal isobaric systems, MDS was performed for 100 ns at room temperature and pressure. An integral step of 2 fs was used for constraining hydrogen bonds in the MDS using the LINCS algorithm. Gmx_MMPBSA (Valdés-Tresanco et al., 2021) was used to implement the free energy of binding between a ligand and protein.


$$\Delta G_{bind}=\Delta G_{complex}-(\Delta G_{receptor}+\Delta G_{ligand})$$

$$=\Delta E_{\text{internal}}+\Delta E_{VDW}+\Delta E_{elec}+\Delta G_{GB}+\Delta G_{SA}$$

The formula above shows that a component known as inner energy contains E_bond_, E_angle_, and E_torsion_. E_VDW_ denotes VDWAALS action, and E_elec_ denotes electrostatic interaction. The free energy of solvation was defined by G_GB_ and G_GA_ together. The solvation energy in polar solutions is G_GB_ while that in nonpolar solutions is G_SA_. Calculations were performed using the GB model (GB = 8) developed by Ukai et al. (2020). The solvent accessibility surface area (SASA) was multiplied by the surface tension of the nonpolar solvent to calculate its free energy of solvation (GSA = 0.0072 × SASA) (Weiser et al., 1999). Because computational resources were limited and precision was low, entropy changes were neglected in this study.

## 3 Results

### 3.1 Network pharmacology results

#### 3.1.1 Active anti-atherosclerotic ingredients and target proteins of VO

Using TCMSP, all the active ingredients in VO were identified. By referring to the pharmacokinetic parameters, 12 components, including quercetin, luteolin, kaempferol, and beta-sitosterol, were screened (**Table 1**). Using the ClassyFire online program, five component categories were identified: six flavonoids (50%), two steroids and steroid derivatives (17%), two prenol lipids (17%), one fatty acyl (8%), and one macrolide and its analogs (8%). Among the identified active ingredients, 1((4aS,6aR,6aS,6bR,8aR,10R,12aR,14bS)-10-hydroxy-2,2,6a,6b,9,9,12a-heptamethyl-1,3,4,5,6,6a,7,8,8a,10,11,12,13,14b-tetradecahydropicene-4a-carboxylic acid) was not involved in the construction of the network diagram, indicating that the efficacy of the active ingredients in VO was not explored.

**Table 1:** Main ingredients in VO and their pharmacological and molecular properties.

Mol ID	Name	Formula	MW (g/mol)	AlogP	Hdon	Hacc	OB (%)	DL	Class
MOL000098	quercetin	C_15_H_10_O_7_	302.25	1.5	5	7	46.43	0.28	Flavonoids
MOL000422	kaempferol	C_15_H_10_O_6_	286.25	1.77	4	6	41.88	0.24	Flavonoids
MOL000006	luteolin	C_15_H_10_O_6_	286.25	2.07	4	6	36.16	0.25	Flavonoids
MOL000358	beta-sitosterol	C_29_H_50_O	414.79	8.08	1	1	36.91	0.75	Steroids and steroid derivatives
MOL000449	Stigmasterol	C_29_H_48_O	412.77	7.64	1	1	43.83	0.76	Steroids and steroid derivatives
MOL005229	Artemetin	C_20_H_20_O_8_	388.4	2.31	1	8	49.55	0.48	Flavonoids
MOL002773	beta-carotene	C_40_H_56_	536.96	12	0	0	37.18	0.58	Prenol lipids
MOL002933	5,7,4’-Trihydroxy-8-methoxyflavone	C_16_H_12_O_6_	300.28	2.32	3	6	36.56	0.27	Flavonoids
MOL008752	Dihydroverticillatine	C_25_H_29_NO_5_	423.55	3.98	2	6	42.69	0.84	Macrolides and analogues
MOL002881	Diosmetin	C_16_H_12_O_6_	300.28	2.32	3	6	31.14	0.27	Flavonoids
MOL005503	Cornudentanone	C_22_H_34_O_5_	378.56	4.97	0	5	39.66	0.33	Fatty acyls
MOL001663	(4aS,6aR,6aS,6bR,8aR,10R,12aR,14bS)-10-hydroxy-2,2,6a,6b,9,9,12a-heptamethyl-1,3,4,5,6,6a,7,8,8a,10,11,12,13,14b-tetradecahydropicene-4a-carboxylic acid	C_30_H_48_O_3_	456.78	6.42	2	3	32.03	0.61	Prenol lipids

MW, molecule weight; ALogP, the real behavior of ionizable compounds in oil–water two-phase at a given PH value; Hdon, hydrogen bond donors; Hacc, hydrogen bond acceptors; Rbon, rotatable bonds; OB, oral bioavailability; DL, drug-likeness.

**Figure 2:**
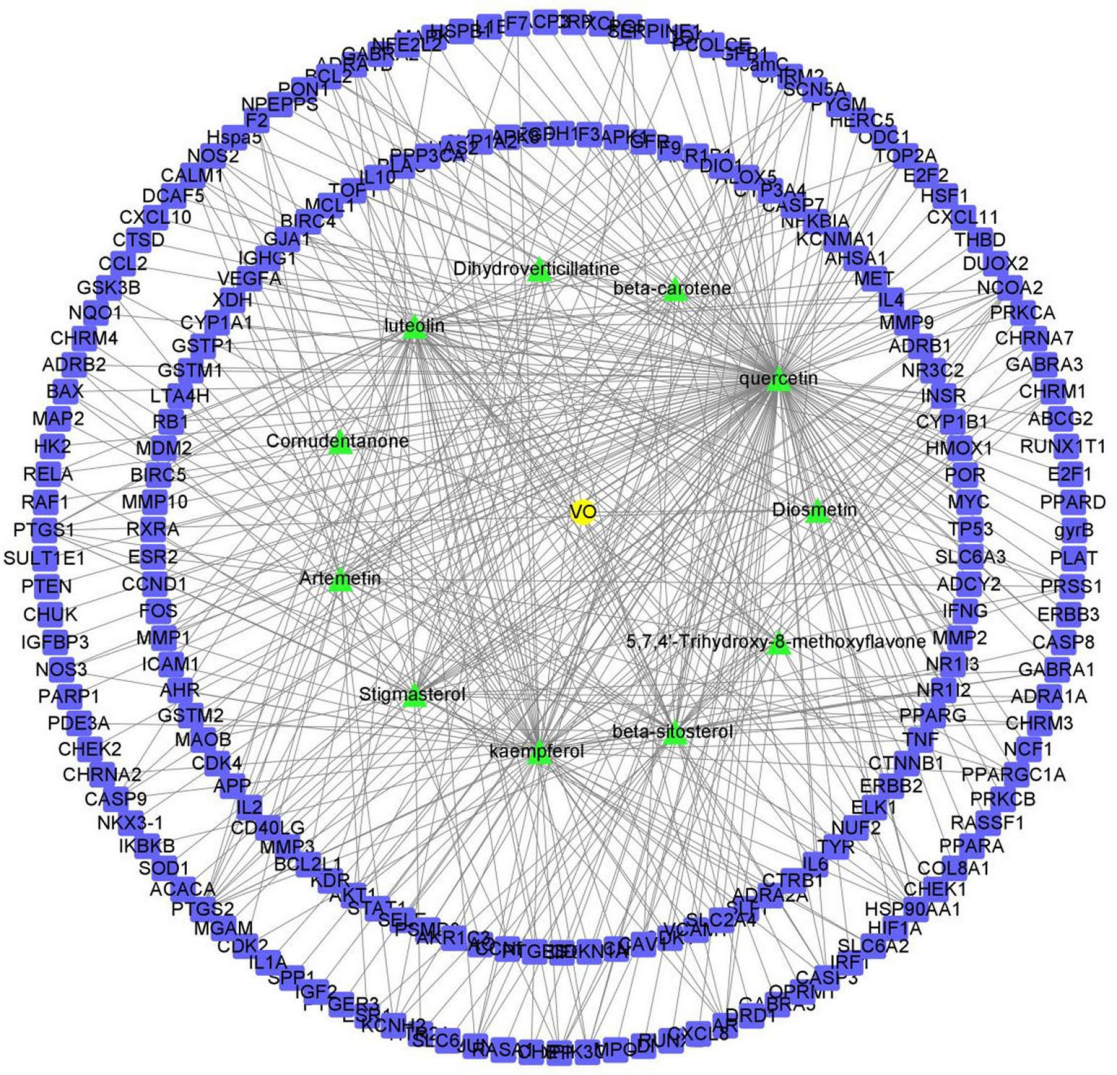
Network of the potential effective ingredients in VO and targets. The green nodes represent the 11 effective ingredients in VO, and the blue nodes represent the 209 corresponding target points of the effective ingredients. There are nodes in the network diagram that represent the potential targets of the genes, and the links between nodes represent the interactions between proteins.

Apart from confirming the targets of the VO ingredients, we identified 209 potential targets using the UniProt database. **Figure 2** demonstrates that quercetin, luteolin, and kaempferol had the largest number of corresponding targets on the list containing the 11 active ingredients. Evidence suggests that these three ingredients in VO are vital for treating ailments.

**Figure 3:**
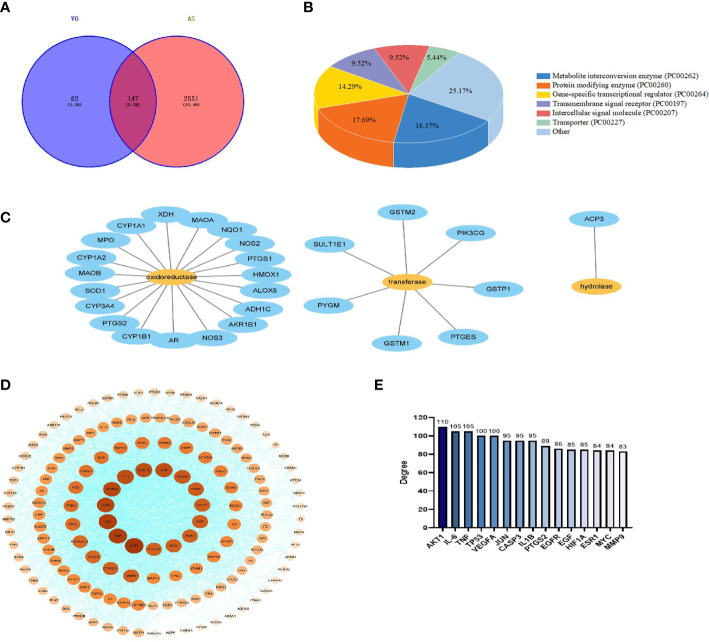
Construction of a PPI network for VO target proteins against AS. **(A)** The TCMSP database was screened, and 209 VO targets were identified. Further, using the DisGeNET, GeneCards, and OMIM databases, 2,698 genes related to AS were identified. VO and AS intersect at 147 points on the Venn diagram. **(B)** Panther classification was used to categorize the target proteins targeted by VO against AS. **(C)** As per Panther classification, the target proteins that modify proteins are classified as enzymes (PC00262). **(D)** PPI network for VO targets against AS. Proteins are represented by nodes, and interactions between targets are represented by edges. The color and degree of a node are correlated, with a darker color indicating a larger node. **(E)** The top 15 core targets were identified based on their degree of importance. Degrees are represented by the number on the vertical axis. Nodes are ranked based on the number of nodes that are directly connected to them. A node with a higher degree is considered an important node.

To identify the targets of AS, keywords were entered into the GeneCards, DisGeNET, and OMIM databases; subsequently, 4,848, 2,044, and three targets, respectively, were screened. After combining the data from the three databases and removing duplicates, 2,698 AS targets were identified. To further determine the effectiveness of both VO and AS, a Venn diagram was constructed to summarize 147 VO-related anti-atherosclerotic targets (**Figure 3A**). Detailed information about these targets is provided in **Table 2**.

**Table 2:** A list of VO targets against AS.

Number	Gene ID	Gene Symbol	Description
1	9429	ABCG2	ATP-binding cassette sub-family G member 2
2	31	ACACA	Acetyl-CoA carboxylase 1
3	43	ACHE	Acetylcholinesterase
4	55	ACP3	Prostatic acid phosphatase
5	126	ADH1C	Alcohol dehydrogenase 1C
6	148	ADRA1A	Alpha-1A adrenergic receptor
7	34971	ADRB1	Beta-1 adrenergic receptor
8	154	ADRB2	Beta-2 adrenergic receptor
9	196	AHR	Aryl hydrocarbon receptor
10	10598	AHSA1	Activator of 90 kDa heat shock protein ATPase homolog 1
11	231	AKR1B1	Aldose reductase
12	207	AKT1	RAC-alpha serine/threonine-protein kinase
13	240	ALOX5	Arachidonate 5-lipoxygenase
14	351	APP	Amyloid beta A4 protein
15	367	AR	Androgen receptor
16	581	BAX	Apoptosis regulator BAX
17	596	BCL2	Apoptosis regulator Bcl-2
18	598	BCL2L1	Bcl-2-like protein 1
19	332	BIRC5	Baculoviral IAP repeat-containing protein 5
20	836	CASP3	Caspase-3
21	842	CASP9	Caspase-9
22	857	CAV1	Caveolin-1
23	6347	CCL2	C-C motif chemokine 2
24	595	CCND1	G1/S-specific cyclin-D1
25	959	CD40LG	CD40 ligand
26	1026	CDKN1A	Cyclin-dependent kinase inhibitor 1
27	11200	CHEK2	Serine/threonine-protein kinase Chk2
28	1128	CHRM1	Muscarinic acetylcholine receptor M1
29	1131	CHRM3	Muscarinic acetylcholine receptor M3
30	1139	CHRNA7	Neuronal acetylcholine receptor protein, alpha-7 chain
31	1147	CHUK	Inhibitor of nuclear factor kappa-B kinase subunit alpha
32	1295	COL8A1	Collagen alpha-1(III) chain
33	1401	CRP	C-reactive protein
34	1499	CTNNB1	Catenin beta-1
35	1509	CTSD	Cathepsin D
36	3627	CXCL10	C-X-C motif chemokine 10
37	2920	CXCL2	C-X-C motif chemokine 2
38	3576	CXCL8	Interleukin-8
39	1543	CYP1A1	Cytochrome P450 1A1
40	1544	CYP1A2	Cytochrome P450 1A2
41	1545	CYP1B1	Cytochrome P450 1B1
42	1576	CYP3A4	Cytochrome P450 3A4
43	1803	DPP4	Dipeptidyl peptidase IV
44	1869	E2F1	Transcription factor E2F1
45	1950	EGF	Pro-epidermal growth factor
46	1956	EGFR	Epidermal growth factor receptor
47	2002	ELK1	ETS domain-containing protein Elk-1
48	2064	ERBB2	Receptor tyrosine-protein kinase erbB-2
49	2099	ESR1	Estrogen receptor
50	2100	ESR2	Estrogen receptor beta
51	2147	F2	Thrombin
52	2152	F3	Tissue factor
53	2155	F7	Coagulation factor VII
54	2158	F9	Coagulation factor Xa
55	2353	FOS	Proto-oncogene c-Fos
56	2697	GJA1	Gap junction alpha-1 protein
57	2932	GSK3B	Glycogen synthase kinase-3 beta
58	2944	GSTM1	Glutathione S-transferase Mu 1
59	2946	GSTM2	Glutathione S-transferase Mu 2
60	2950	GSTP1	Glutathione S-transferase P
61	3091	HIF1A	Hypoxia-inducible factor 1-alpha
62	3162	HMOX1	Heme oxygenase 1
63	3320	HSP90AA1	Heat shock protein HSP 90
64	3315	HSPB1	Heat shock protein beta-1
65	3356	HTR2A	5-hydroxytryptamine 2A receptor
66	3383	ICAM1	Intercellular adhesion molecule 1
67	3458	IFNG	Interferon gamma
68	3481	IGF2	Insulin-like growth factor II
69	3486	IGFBP3	Insulin-like growth factor-binding protein 3
70	147100	IGHG1	Ig gamma-1 chain C region
71	3551	IKBKB	Inhibitor of nuclear factor kappa-B kinase subunit beta
72	3586	IL10	Interleukin-10
73	3552	IL1A	Interleukin-1 alpha
74	3553	IL1B	Interleukin-1 beta
75	3558	IL2	Interleukin-2
76	3565	IL4	Interleukin-4
77	3569	IL6	Interleukin-6
78	3643	INSR	Insulin receptor
79	3659	IRF1	Interferon regulatory factor 1
80	3725	JUN	Transcription factor AP-1
81	3757	KCNH2	Potassium voltage-gated channel subfamily H member 2
82	3791	KDR	Vascular endothelial growth factor receptor 2
83	4048	LTA4H	Leukotriene A-4 hydrolase
84	4128	MAOA	Amine oxidase [flavin-containing] A
85	4129	MAOB	Amine oxidase [flavin-containing] B
86	5594	MAPK1	Mitogen-activated protein kinase 1
87	1432	MAPK14	Mitogen-activated protein kinase 14
88	5599	MAPK8	Mitogen-activated protein kinase 8
89	4193	MDM2	E3 ubiquitin-protein ligase Mdm2
90	4233	MET	Hepatocyte growth factor receptor
91	4312	MMP1	Interstitial collagenase
92	4319	MMP10	Stromelysin-2
93	4313	MMP2	72 kDa type IV collagenase
94	4314	MMP3	Stromelysin-1
95	4318	MMP9	Matrix metalloproteinase-9
96	4353	MPO	Myeloperoxidase
97	4609	MYC	Myc proto-oncogene protein
98	653361	NCF1	Neutrophil cytosol factor 1
99	10499	NCOA2	Nuclear receptor coactivator 2
100	4780	NFE2L2	Nuclear factor erythroid 2-related factor 2
101	4792	NFKBIA	NF-kappa-B inhibitor alpha
102	4843	NOS2	Nitric oxide synthase, inducible
103	4846	NOS3	Nitric-oxide synthase, endothelial
104	1728	NQO1	NAD(P)H dehydrogenase [quinone] 1
105	8856	NR1I2	Nuclear receptor subfamily 1 group I member 2
106	9970	NR1I3	Nuclear receptor subfamily 1 group I member 3
107	4306	NR3C2	Mineralocorticoid receptor
108	142	PARP1	Poly [ADP-ribose] polymerase 1
109	5111	PCNA	Proliferating cell nuclear antigen
110	5294	PIK3CG	Phosphatidylinositol-4,5-bisphosphate 3-kinase catalytic subunit, gamma isoform
111	5327	PLAT	Tissue-type plasminogen activator
112	5328	PLAU	Urokinase-type plasminogen activator
113	5444	PON1	Serum paraoxonase/arylesterase 1
114	5465	PPARA	Peroxisome proliferator-activated receptor alpha
115	5467	PPARD	Peroxisome proliferator-activated receptor delta
116	5468	PPARG	Peroxisome proliferator-activated receptor gamma
117	10891	PPARGC1A	Peroxisome proliferator activated receptor gamma
118	5578	PRKCA	Protein kinase C alpha type
119	5579	PRKCB	Protein kinase C beta type
120	5728	PTEN	Phosphatidylinositol-3,4,5-trisphosphate 3-phosphatase and dual-specificity protein phosphatase PTEN
121	9536	PTGES	Prostaglandin E synthase
122	5742	PTGS1	Prostaglandin G/H synthase 1
123	5743	PTGS2	Prostaglandin G/H synthase 2
124	5837	PYGM	Glycogen phosphorylase, muscle form
125	5894	RAF1	RAF proto-oncogene serine/threonine-protein kinase
126	5921	RASA1	Ras GTPase-activating protein 1
127	5925	RB1	Retinoblastoma-associated protein
128	5970	RELA	Transcription factor p65
129	860	RUNX2	Runt-related transcription factor 2
130	6256	RXRA	Retinoic acid receptor RXR-alpha
131	6331	SCN5A	Sodium channel protein type 5 subunit alpha
132	6401	SELE	E-selectin
133	5054	SERPINE1	Plasminogen activator inhibitor 1
134	6517	SLC2A4	Solute carrier family 2, facilitated glucose transporter member 4
135	6531	SLC6A4	Sodium-dependent serotonin transporter
136	6647	SOD1	Superoxide dismutase [Cu-Zn]
137	6696	SPP1	Osteopontin
138	6772	STAT1	Signal transducer and activator of transcription 1-alpha/beta
139	6783	SULT1E1	Estrogen sulfotransferase
140	7040	TGFB1	Transforming growth factor beta-1
141	7056	THBD	Thrombomodulin
142	7124	TNF	Tumor necrosis factor
143	7150	TOP1	DNA topoisomerase 1
144	7157	TP53	Cellular tumor antigen p53
145	7412	VCAM1	Vascular cell adhesion protein 1
146	7422	VEGFA	Vascular endothelial growth factor A
147	7498	XDH	Xanthine dehydrogenase/oxidase

To confirm the function of the potential VO targets, the Panther classification system was used to classify the 147 VO targets against AS. These targets were classified into seven classes according to their cellular functions: metabolite interconversion enzyme (PC00262, 18.37%), protein-modifying enzyme (PC00260), gene-specific transcriptional regulator (PC00264), transmembrane signal receptor (PC00197), intercellular signal molecule (PC00207), transporter (PC00227), and others; of these, metabolite interconversion enzymes (18.37%) were the most abundant class (**Figure 3B**), followed by oxidoreductases (PC00176, 70.37%), transferases (PC00220, 25.93%), hydrolases (PC00121, 3.70%) (**Figure 3C**).

Using the STRING database, 147 AS and VO intersection targets were determined, followed by mapping in Cytoscape. The degree of a node indicates its importance based on the number of edges connecting it to other nodes in the PPI network diagram. A PPI network comprising 145 nodes, 2,988 edges, and a degree of average quality of 40.9 was constructed (**Figure 3D**). The potential targets were identified as AKT1, IL-6, TNF, TP53, VEGFA, JUN, CASP3, IL1B, PTGS2, EGFR, EGF, HIF1A, ESR1, MYC, and MMP9, depending on the degree of the node. Among them, AKT1, IL-6, and TNF were the first three targets with the highest correlation, with degree values of 110, 105, and 105, respectively (**Figure 3E**). These results indicate that the ingredients in VO are effective against AS *via* multiple targets and many biological mechanisms.

#### 3.1.2 Target–AS target network construction for VO ingredients

**Figure 4:**
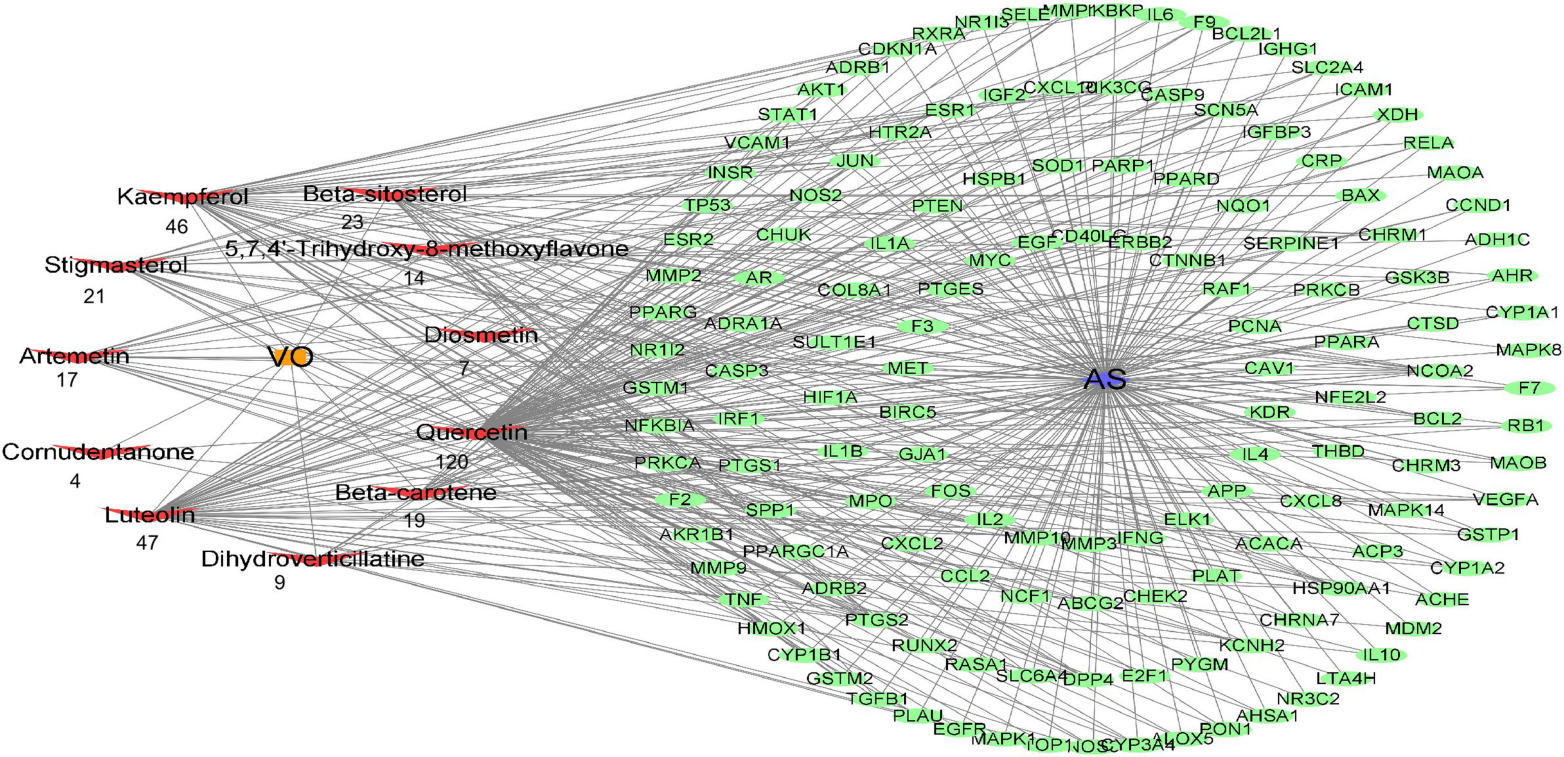
Construction of the drug–target–disease network. Each red node represents an effective ingredient, and the number beneath each node indicates how effective that ingredient is against AS. Cytoscape represents the green nodes as potential targets for VO and AS.

To determine the effective VO ingredients for treating AS, 147 possible targets and 11 effective VO ingredients were selected for constructing an effective ingredient network for VO and AS (**Figure 4**). Degree refers to how many edges are connected to a node; therefore, a higher degree value suggests the greater importance of the node. Notably, all these ingredients were associated with multiple targets, resulting in 327 ingredient–target connections for 11 effective VO ingredients and 147 targets. In total, 29.7 targets were identified per ingredient, and the average number of components per target was 2.2. These findings indicate that VO possesses the multicomponent and multitarget characteristics of traditional Chinese medicines. Among these bioactive ingredients, quercetin (degree = 120) had the most targets, followed by luteolin (degree = 47), kaempferol (degree = 46), beta-sitosterol (degree = 23), stigmasterol (degree = 21), beta-carotene (degree = 19), artemetin (degree = 17), 5,7,4’-trihydroxy-8-methoxyflavone (degree = 14), dihydroverticillatine (degree = 9), diosmetin (degree = 7), and cornudentanone (degree = 4). Taken together, these findings suggest that quercetin, luteolin, and kaempferol are probably the most important VO ingredients needed for treating AS.

#### 3.1.3 Potential synergistic mechanism of VO against AS

**Figure 5:**
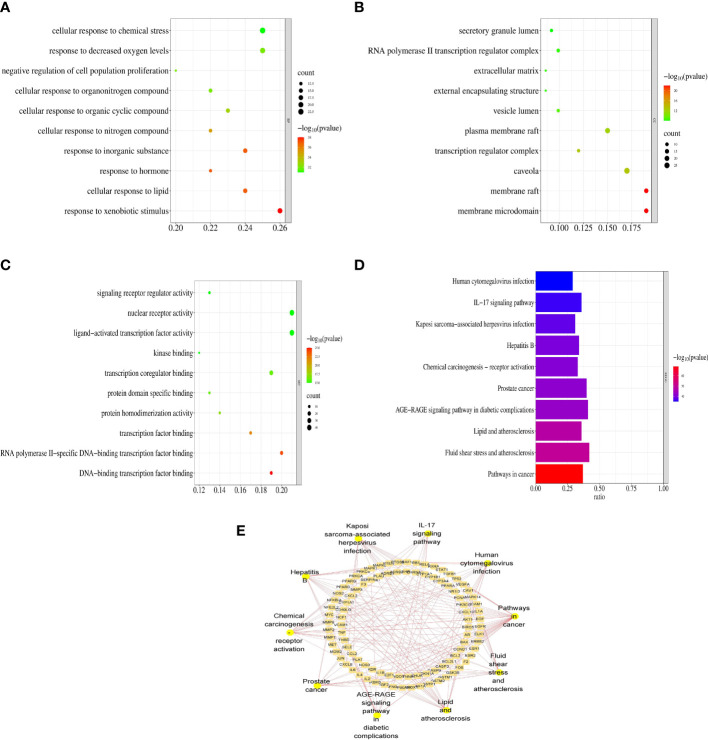
Functional enrichment analyses of the target proteins of VO against AS. **(A)** GO enrichment analysis reveals the top 10 biological processes. **(B)** A bubble chart illustrating the top 10 biological processes that are involved in cell components. **(C)** A bubble chart of the top 10 molecular functions derived from GO enrichment analysis. Rich factor is indicated on the X-axis, bubble size indicates target counts enriched in terms, and color indicates the significance as p-value. According to the order of the p-values from small to large, the top 10 vital GO terms were selected from the VO targets against AS. **(D)** VO anti-AS targets were analyzed for KEGG pathway enrichment. According to the order of the p-value from small to large, the 10 most significant KEGG pathways were chosen. **(E)** The top 10 pathways, along with the targets associated with them, as shown in the Cytoscape software.

To investigate the synergistic mechanisms of the main VO ingredients against AS, the DAVID database was used to assess the 147 VO targets involved in AS pathology *via* GO enrichment analysis. **Figure 5A** presents the top 10 enriched GO terms based on their adjusted p-values. The major focus of BP was response to xenobiotic stimulus (GO:0009410), cellular response to lipid (GO:0071396), response to hormone (GO:0009725), and so on. Most CC research focused on the membrane microdomain (GO:0098857), membrane raft (GO:0045121), and caveola (GO:0005901) in **Figure 5B**. In **Figure 5C**, MF was mainly focused on DNA-binding transcription factor binding (GO:0140297), RNA polymerase II-specific DNA-binding transcription factor binding (GO:0061629), and transcription factor binding (GO:0008134). These findings suggest that VO plays an essential biological role in AS development.

To identify KEGG pathways related to effective VO ingredients against AS targets, the Metascape database, which contains 147 targets, was enriched for KEGG pathway enrichment analysis. The top 10 pathways with the highest significance are shown in **Figure 5D**. The main pathways involved in cancer (hsa05200), fluid shear stress and atherosclerosis (hsa05418), lipids and atherosclerosis (hsa05417), the AGE-RAGE signaling pathway in diabetic complications (hsa04933), and prostate cancer (hsa05215), along with the top 10 pathways along with the targets associated with them (**Figure 5E**). **Table 3** provides a detailed report of the KEGG pathway enrichment analysis. Based on these findings, VO exerts its anti-atherosclerotic effects *via* multiple pathways.

**Table 3:** Top 10 terms in the KEGG pathways associated with AS enriched in VO.

Pathway	Degree	Gene
Pathways in cancer	62	AKT1|BIRC5|AR|BAX|CCND1|BCL2|BCL2L1|CASP3|CASP9|CDKN1A|CHUK|CTNNB1|NQO1|E2F1|EGF|EGFR|ELK1|ERBB2|ESR1|ESR2|F2|FOS|GSK3B|GSTM1|GSTM2|GSTP1|HIF1A|HMOX1|HSP90AA1|IFNG|IGF2|IKBKB|IL2|IL4|IL-6|CXCL8|JUN|MDM2|MET|MMP1|MMP2|MMP9|MYC|NFE2L2|NFKBIA|NOS2|PPARD|PPARG|PRKCA|PRKCB|MAPK1|MAPK8|PTEN|PTGS2|RAF1|RB1|RELA|RXRA|STAT1|TGFB1|TP53|VEGFA
Lipid and AS	38	AKT1|BAX|BCL2|BCL2L1|CASP3|CASP9|CD40LG|CHUK|MAPK14|CYP1A1|FOS|CXCL2|GSK3B|HSP90AA1|ICAM1|IKBKB|IL1B|IL-6|CXCL8|JUN|MMP1|MMP3|MMP9|NFE2L2|NFKBIA|NOS3|PPARG|PRKCA|MAPK1|MAPK8|RELA|RXRA|CCL2|SELE|TNF|TP53|VCAM1|NCF1
Fluid shear stress and AS	35	AKT1|BCL2|CAV1|CHUK|MAPK14|CTNNB1|NQO1|FOS|GSTM1|GSTM2|GSTP1|HMOX1|HSP90AA1|ICAM1|IFNG|IKBKB|IL1A|IL1B|JUN|KDR|MMP2|MMP9|NFE2L2|NOS3|PLAT|MAPK8|RELA|CCL2|SELE|THBD|TNF|TP53|VCAM1|VEGFA|NCF1
Chemical carcinogenesis - receptor activation	34	ADRB1|ADRB2|AHR|AKT1|BIRC5|AR|CCND1|BCL2|CHRNA7|CYP1A1|CYP1A2|CYP1B1|CYP3A4|E2F1|EGF|EGFR|ESR1|ESR2|FOS|GSTM1|GSTM2|HSP90AA1|JUN|MYC|PPARA|PRKCA|PRKCB|MAPK1|RAF1|RB1|RELA|RXRA|VEGFA|NR1I3
Hepatitis B	31	AKT1|BIRC5|BAX|BCL2|CASP3|CASP9|CDKN1A|CHUK|MAPK14|E2F1|ELK1|FOS|IKBKB|IL-6|CXCL8|JUN|MMP9|MYC|NFKBIA|PCNA|PRKCA|PRKCB|MAPK1|MAPK8|RAF1|RB1|RELA|STAT1|TGFB1|TNF|TP53
Kaposi sarcoma-associated herpesvirus infection	31	AKT1|BAX|CCND1|CASP3|CASP9|CDKN1A|CHUK|MAPK14|CTNNB1|E2F1|FOS|CXCL2|GSK3B|HIF1A|ICAM1|IKBKB|IL-6|CXCL8|JUN|MYC|NFKBIA|PIK3CG|MAPK1|MAPK8|PTGS2|RAF1|RB1|RELA|STAT1|TP53|VEGFA
Human cytomegalovirus infection	31	AKT1|BAX|CCND1|CASP3|CASP9|CDKN1A|CHUK|MAPK14|CTNNB1|E2F1|EGFR|ELK1|GSK3B|IKBKB|IL1B|IL-6|CXCL8|MDM2|MYC|NFKBIA|PRKCA|PRKCB|MAPK1|PTGS2|RAF1|RB1|RELA|CCL2|TNF|TP53|VEGFA
AGE-RAGE signaling pathway in diabetic complications	29	AKT1|BAX|CCND1|BCL2|CASP3|MAPK14|F3|ICAM1|IL1A|IL1B|IL-6|CXCL8|JUN|MMP2|NOS3|SERPINE1|PRKCA|PRKCB|MAPK1|MAPK8|RELA|CCL2|SELE|STAT1|TGFB1|THBD|TNF|VCAM1|VEGFA
Prostate cancer	28	AKT1|AR|CCND1|BCL2|CASP9|CDKN1A|CHUK|CTNNB1|E2F1|EGF|EGFR|ERBB2|GSK3B|GSTP1|HSP90AA1|IKBKB|MDM2|MMP3|MMP9|NFKBIA|PLAT|PLAU|MAPK1|PTEN|RAF1|RB1|RELA|TP53
IL-17 signaling pathway	25	CASP3|CHUK|MAPK14|FOS|CXCL2|GSK3B|HSP90AA1|IFNG|IKBKB|IL1B|IL4|IL-6|CXCL8|CXCL10|JUN|MMP1|MMP3|MMP9|NFKBIA|MAPK1|MAPK8|PTGS2|RELA|CCL2|TNF

### 3.2 Molecular docking

To validate the binding of VO ingredients to the molecules involved in AS pathology, molecular docking was performed using Smina software. **Table 4** presents the docking scores between quercetin, luteolin, and kaempferol and the main targets (AKT1, IL-6, and TNF). Lower scores were determined by better docking effects. In terms of affinity, quercetin exhibited the strongest affinity with AKT1, IL-6, and TNF-α, with scores of −9.6, −8.9, and −8.8 kcal/mol, respectively. The binding scores of luteolin to AKT1, IL-6, and TNF-α were −9.5, −8.4, and −7.3 kcal/mol and those of kaempferol were −9.0, −8.4, and −7.9 kcal/mol.

**Table 4:** Binding energies of the molecular docking of VO with targets.

	kaempferol	luteolin	Quercetin
AKT1	−9.0 kcal/mol	−9.5 kcal/mol	−9.6 kcal/mol
TNF-α	−7.9 kcal/mol	−7.3 kcal/mol	−8.8 kcal/mol
IL-6	−8.4 kcal/mol	−8.4 kcal/mol	−8.9 kcal/mol

Because the proteins are structures generated by AlphaFold2, the default protein center of gravity is at the origin of spatial coordinates.

**Figure 6:**
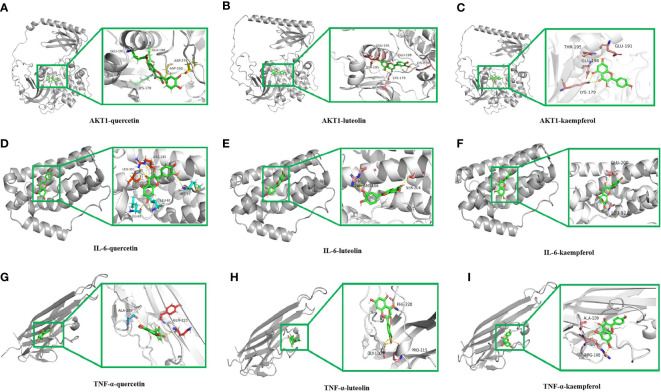
Molecular docking of the main ingredients in VO with AKT1, IL-6, and TNF-α. **(A)** 3D binding posture schematic diagram of AKT1 and quercetin. **(B)** 3D binding posture schematic diagram of AKT1 and luteolin. **(C)** 3D binding posture schematic diagram of AKT1 and kaempferol. **(D)** 3D binding posture schematic diagram of IL-6 and quercetin. **(E)** 3D binding posture schematic diagram of IL-6 and luteolin. **(F)** 3D binding posture schematic diagram of IL-6 and kaempferol. **(G)** 3D binding posture schematic diagram of TNF-α and quercetin. **(H)** 3D binding posture schematic diagram of TNF-α and luteolin. **(I)** 3D binding posture schematic diagram of TNF-α and kaempferol. (TNF-α was selected for docking with TNF.).

As shown in **Figure 6**, the binding pockets of AKT1, IL-6, and TNF-α were tightly bound by kaempferol, luteolin, and quercetin, which were stabilized by hydrogen bonds. Four amino acid residues, namely, GLU191, GLU198, LYS179, and ASP292 form hydrogen bonds with quercetin in AKT1, whereas ASP274 plays a hydrophobic role (**Figure 6A**). Furthermore, five amino acid residues, namely, GLU191, GLU198, LYS179, LYS276 and THR195, formed hydrogen bonds with luteolin in AKT1 (**Figure 6B**), and four amino acid residues, namely, GLU191, GLU198, LYS179, and THR195, in AKT1 formed hydrogen bonds with kaempferol (**Figure 6C**). IL-6 contains the amino acid residues LEU90 and ARG196 that form multiple binding sites for quercetin; these are surrounded by hydrophobic forces such as LEU193, LEU92, and MET95 (**Figure 6D**). Luteolin forms potential interactions with IL-6 *via* the amino acid residues ARG196 and SER204 and two hydrogen bonds (**Figure 6E**). The amino acid residues GLU200 and LEU92 in IL-6 form several hydrogen bonds with kaempferol (**Figure 6F**). The amino acid residue ALA109 in TNF-α forms a hydrogen bond with quercetin, whereas GLN225 plays a hydrophobic role in TNF-α (**Figure 6G**). Moreover, luteolin formed potential interactions with the amino acid residues PHE220 and PRO215 of TNF-α *via* two hydrogen bonds, with the amino acid residue GLY100 playing a hydrophobic role (**Figure 6H**). The amino acid residue ALA109 in TNF-α formed multiple binding locations with quercetin, whereas the amino acid residue ARG108 played a hydrophobic role (**Figure 6I**). All binding positions are shown in **Supplementary Figure 1**. Taken together, these findings suggest that the key ingredients in VO strongly bind to targets related to the lipid and AS pathways.

### 3.3 MDS

**Figure 7:**
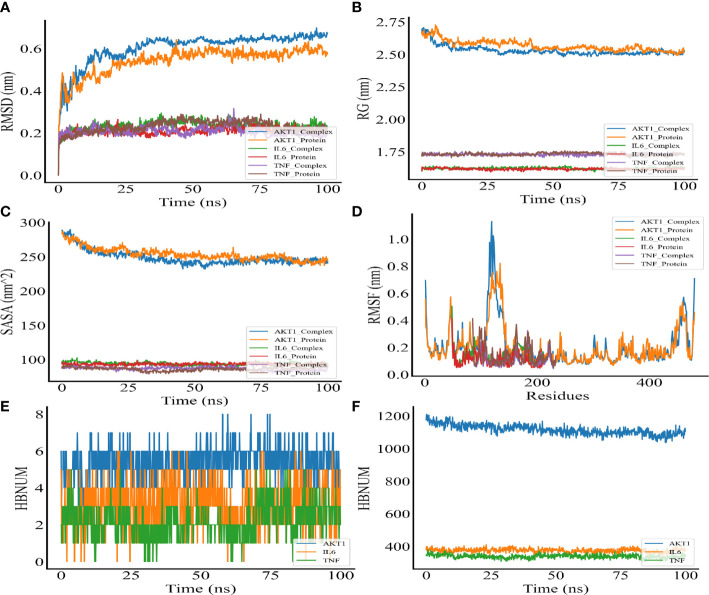
Molecular dynamic simulation. **(A)** RMSD of the complex MDS. **(B)** Rg variation diagram of the complex MDS. **(C)** SASA of the complex MDS. **(D)** RMSF of the complex MDS is displayed in the high fluctuation range of the surface. **(E)** HBNUM in the complex MDS. **(F)** Number of hydrogen bonds between proteins and water (HBNUM) (protein represents a blank protein, and complex represents core target proteins and core ligands).

**Figure 8:**
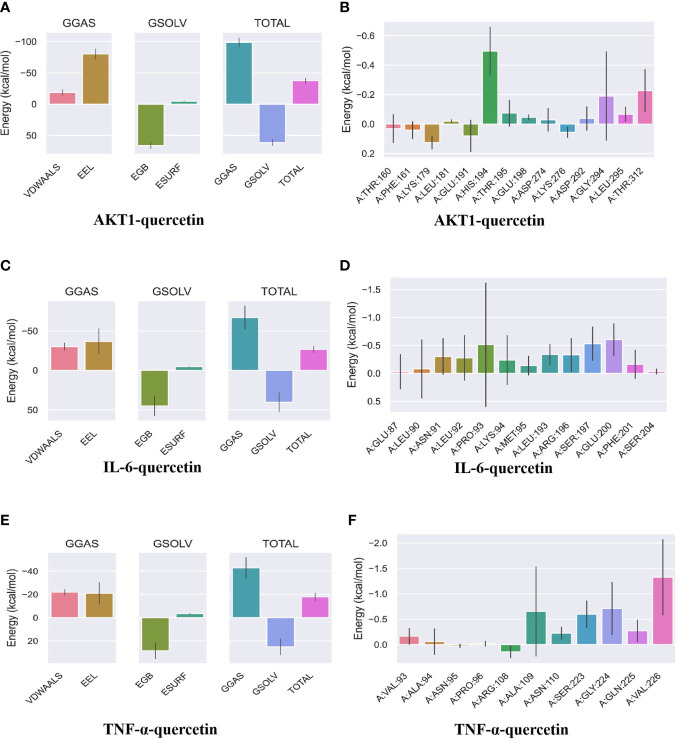
Binding free energy analysis. **(A)** Energy attribute breakdown diagram for AKT1–quercetin. **(B)** Energy amino acid decomposition diagram for AKT1–quercetin. **(C)** Energy attribute breakdown diagram for IL-6-quercetin. **(D)** Energy amino acid decomposition diagram for IL-6–quercetin. **(E)** Energy attribute breakdown diagram for TNF-α–quercetin. **(F)** Energy amino acid decomposition diagram for TNF-α–quercetin. A method based on MM-GBSA was used to calculate free energy and decompose residues. VDWAALS, van der Waals energy; EEL, polar solvation energy; EGB, polar solvation energy; ESURF, nonpolar solvation energy; GGAS, total gas phase free energy; GSOLV, Total solvation free energy; TOTAL, GSOLV + GGAS.

MDS was performed to further verify the binding between quercetin and AKT1, IL-6, and TNF-α. Root-mean-square deviation (RMSD) was used as a measure of the system’s stability. During the six dynamic simulation sets, it fluctuated slightly but remained stable at 0.20–0.25 nm/0.5–0.6 nm after simulation for 25 ns (**Figure 7A**). The radius of gyration (Rg) was used as a metric for determining the tightness of the architecture. It is an estimation of the distance between the center of mass of a protein atom and its terminal atoms. The fluctuation amplitude (degree of fluctuation) and RMSD of the six simulation groups were consistent, and the value of the AKT1 system was higher for both RMSD and Rg (**Figure 7B**). When a solvent molecule seeks the VDWAALS surface of a protein, it monitors a portion of the protein’s surface known as SASA. SASA steadily decreased from 0 to 100 ns, indicating that the protein was constricted/closed and that its hydrophilic surface area had decreased (**Figure 7C**). The root-mean-square function (RMSF) is used to observe the allotropy of the local site of the system during simulations (**Figure 7D**). Because hydrogen bonds contributed to the major interactions for complex formation and stability, they exerted a significant effect in determining the stability of the protein–ligand complex. **Figures 7E, F** illustrate the number of hydrogen bonds for small molecules and proteins and proteins and systems, respectively. AKT1 had four to six contacts with small molecules, IL-6 had two to five contacts, and TNF-α had one to three contacts. First, the binding free energies of the protein–ligand complex were calculated, followed by comparisons between bound and unbound solvated molecules. To achieve this, different conformations of the same molecule were compared in terms of their free energies (**Figure 8**). Solvation free energy can be determined by studying the interactions between polar and nonpolar residues of a protein. Using MDS, we found that the total free energy was negative after determining the change in free energy. This could have been because of interactions between the protein and small molecules. The affinity of the protein–ligand of the AKT1 system was −28.093 kcal/mol (**Figure 8A**), that of the IL-6 system was −26.52 kcal/mol (**Figure 8C**), and that of the TNF-α system was −17.77 kcal/mol (**Figure 8E**). The decomposition diagram of free energy residues can be used to understand which amino acid residues contribute to the binding during ligand simulation. In the AKT1 system, HIS194, GLY198, GLY294, and THR312 positively contributed to free energy, which was conducive to binding, whereas GLU191 was not conducive to binding (**Figure 8B**). Further, LEU90, PRO93, ARG196, and GLU200 positively contributed to free energy in the IL-6 system, which was conducive to binding (**Figure 8D**). Lastly, in the AKT1 system, ALA109, SER223, GLY224, and ALA226 positively contributed to free energy, which was conducive to binding (**Figure 8F**). Taken together, the results suggest that quercetin stably binds with AKT1, IL-6, and TNF-α, making it a promising agent for treating AS.

## 4 Discussion

Traditional Chinese medicines can be used to treat diseases involving multiple targets and components. They exhibit the ability to inhibit endothelial dysfunction, platelet activation, lipid peroxidation, ROS production, and macrophage-induced AS (Kirichenko et al., 2020). However, the study of traditional Chinese medicines is extremely challenging owing to their complex ingredients (Han et al., 2020). A computer-aided drug design approach can be used to gain an in-depth and systematic understanding of the effectiveness and functions of the ingredients in traditional Chinese medicines (Man and Sai, 2022). Previous studies have reported the various pharmacological effects of VO, including regulating lipid metabolism, scavenging free radicals, inhibiting inflammation, and preventing tumors (Li et al., 2022). Therefore, in the present study, we comprehensively and systematically predicted the herb–ingredient–target and gene–pathway interactions for VO against AS using a computer-aided drug design approach involving network pharmacology, molecular docking, and MDS.

Twelve active ingredients were screened according to pharmacokinetic parameters, including flavonoids, steroids and steroid derivatives, prenol lipids, fatty acyls, and macrolides and analogs; this is in agreement with the findings of previous studies (El-Hela et al., 2010; Gibitz-Eisath et al., 2018). Although VO has 12 active ingredients, their action targets remain unknown, indicating that their efficacy has not yet been investigated. In our study, we identified 11 active ingredients in VO with 147 potential targets. Among the main ingredients screened, flavonoids, including quercetin, luteolin, and kaempferol, and steroids and steroid derivatives, including beta-sitosterol and stigmasterol, had high degrees of nodes, and quercetin had the most anti-atherosclerotic targets, with 120 core targets. Evidence suggests that quercetin suppresses the PI3K/AKT pathway *via* the lipid and atherosclerotic pathways, inhibiting downstream NF-KB and reducing inflammatory factors (Xu et al., 2018). Further, quercetin inhibits endothelial dysfunction in AS by reducing HOCl production by MPO/NADPH oxidase (Li et al., 2023). Several studies have demonstrated that luteolin prevents AS by regulating oxidative stress, reducing triglyceride and low-density lipoprotein (LDL) cholesterol levels, and inhibiting plaque development (Makino et al., 2016; Li et al., 2018; Ding et al., 2019). In atherosclerotic ApoE^−/−^ mice, kaempferol targeted the plaque areas and inhibited macrophage-mediated inflammation, accompanied by a decrease in TNF-α proinflammatory cytokines and repolarization of M1 to M2 macrophages (Jianing et al., 2022). A study reported the antioxidant, anti-inflammatory, and cardioprotective effects of kaempferol and suggested its potential as a drug candidate for treating and preventing AS (Chen et al., 2022b). Beta-sitosterol can effectively reduce the production of the intestinal microbial metabolite trimethylamine to ameliorate atherosclerotic plaques in AS mice (Wu et al., 2022). Stigmasterol activates the nuclear receptor LXR to promote cholesterol secretion *via* the intestinal tract and inhibits the expression of proinflammatory mediators such as IL-6, IL-1β, COX-2, and TNF-α to ameliorate AS (Lifsey et al., 2019). Taken together, these findings suggest the anti-atherosclerotic effect of VO, with quercetin, luteolin, and kaempferol as the main ingredients.

In the PPI network, AKT1, IL-6, and TNF-α were chosen as potential targets owing to their high degree of nodality. The degree of a node is determined by the number of nodes directly linked to it. In general, nodes with a higher degree are considered to be more important than those with a lower degree (Zeng et al., 2021). AKT1 is an AKT kinase that regulates cell proliferation and growth and has an antiapoptotic function; its mechanism involves the PI3K/AKT signaling pathway (Meng et al., 2021). The PI3K/AKT pathway is implicated in AS pathogenesis because it participates in lipid metabolism, smooth muscle and fibrocyte proliferation, and collagen production in the arterial wall (Chen et al., 2009). Study has shown that the administration of *Astragalus mongholicus* extract helped improve the lipid profile in high-fat diet-induced mice by lowering adipogenesis, increasing lipolysis and lipid β-oxidation, downregulating AKT1 and CCND1 in the liver, and upregulating VEGFA and ESR1 in the adipose tissue and liver; these findings suggest that AKT1 is an important target for treating AS (Wang et al., 2022). NF-KB, as a vital effector downstream of the PI3K/AKT signaling pathway, promotes the phosphorylation of NF-KB inhibitory protein A (inhibitory subunit alpha of NF-KB); as a result, it dissociates from NF-KB and induces several inflammatory cytokines, including IL-6 and TNF-α, resulting in an inflammatory response (Song et al., 2018). IL-6 and TNF-α induce lipolysis and inhibit lipogenesis, whereas chemokines mediate the recruitment of macrophages and monocytes from the adipose tissue, thereby increasing the production of inflammatory factors to impair fat homeostasis (Chen et al., 2009; Hashizume and Mihara, 2011). A study has shown that TNF-α can participate in lipid metabolism by mediating the role of LXRα in regulating cholesterol efflux (Ikhlef et al., 2016). Overall, these targets are involved in AS progression, consistent with the findings of existing studies (Meng et al., 2021), and can be used as biomarkers for AS as well as targets for drug action. TNF-α is responsible for AS owing to its role as an inflammatory mediator secreted by macrophages (Businaro et al., 2012). A study found a correlation between atherogenic indexes and both IL-1 and IL-6 as a result of spondyloarthritis, and that serum IL-6 and IL-1 levels contribute to the development of AS (Ben Ali et al., 2021). The above studies suggest that VO may treat AS through these targets.

In our study, GO pathway analysis predicted that BP mainly focused on responses to xenobiotic stimuli, cellular responses to lipids, and responses to hormones. A study has shown that the response to xenobiotic stimuli is involved in AS development. The presence of chronic infectious stimuli may directly activate metabolic pathways (e.g., cholesterol esterification) in susceptible individuals, leading to the formation of foam cells (Conti et al., 2010). AS depends on the cellular response to lipids. In the arterial wall, macrophages play an important role in lipid accumulation and generating foam cells filled with atherosclerotic plaques (Sukhorukov et al., 2020). For the response to hormones, previous studies have reported that glucocorticoids can affect vascular responses by regulating vasoconstriction or vasodilation and are essential for regulating blood pressure and blood flow (Macleod et al., 2021). In terms of hormones, estrogen has positive effects on blood lipids, including increasing high-density lipoprotein levels, reducing LDL and LDL oxidation, and controlling triglyceride levels, thereby improving AS (Dobrescu et al., 2020). KEGG pathway enrichment analysis revealed that the intersection targets were mainly related to pathways in cancer, fluid shear stress and atherosclerosis, and lipid and atherosclerosis. A research study reported that AS, similar to cancer, develops *via* clonal proliferation of altered cells during local tissue damage, inflammation, and genomic instability (Ross et al., 2002). Lipid metabolism is important for the effects of AS. Zhou reported that Danhong injection (DHI) reduced the AS index and plaque areas in high-fat diet-induced atherosclerotic mice and that stimulation of the PI3K/AKT signaling pathway led to DHI inhibition of lipid accumulation by macrophages (Zhou et al., 2019a). Li explored the use of the lipid pathway to treat AS and found that the Qing-Xue-Xiao-Zhi formula inhibited lipid accumulation and inflammation in macrophages, regulated and promoted lipid efflux *via* the TLR4/MyD88/NF-κB pathway, inhibited macrophage-mediated inflammation, and exhibited therapeutic effects on AS (Li et al., 2021). These findings suggest the significant biological role of VO in AS development and that it exerts multiple protective effects on AS.

We found that three key ingredients in VO (quercetin, luteolin, and kaempferol) were linked to three AS target genes (AKT1, IL-6, and TNF-α). We used molecular docking to dock the three active ingredients with the three related potential target proteins (Malik et al., 2023). The negative value of the binding energy of the complex indicates that the energy released by the binding is easier to bind and that the complex is more stable (Alshammari, 2023). Molecular docking revealed that quercetin, luteolin, and kaempferol strongly bind with AKT1, IL-6, and TNF-α, respectively. Compared with other binding energy values, the lowest binding energy of quercetin indicated the highest binding energy for these targets. Based on these findings, the key ingredients in VO that bind to the targets related to lipids and AS may be AKT1, IL-6, and TNF-α.MDS can be used to place ligands and receptors in a system that mimics their natural binding. MDS can illustrate the microscopic evolution of a system at an atomic level and intuitively demonstrate the mechanism and law of experimental phenomena, which are critical for understanding the mechanism of ligand–receptor binding. In our study, MDS revealed that the binding between quercetin and AKT1, IL-6, and TNF-α was stable; however, the binding affinity of quercetin to AKT1 was higher. RMSD can be utilized to evaluate system stability, and Rg is an important index used to evaluate the tightness of the architecture. SASA refers to the solvent-accessible surface area of the protein. RMSF allows observation of the allotropy of the local site as the simulation proceeds (Miraz et al., 2023). The stability of protein ligands is greatly influenced by hydrogen bonding. In our study, the RMSD and Rg of the AKT1 system were higher. AKT1 had four to six contacts with small molecules, which was higher than that of IL-6 and TNF-α, further indicating that quercetin is strongly bound to AKT1. Analysis of free energy revealed that AKT1, IL-6, and TNF-α have the same positive and negative sign distribution. However, the affinity of AKT1 was −28.093 kcal/mol, which was stronger than that of IL-6 and TNF-α. This finding once again demonstrates that quercetin was strongly bound to AKT1. VDWAALS energy is used to calculate the total gas phase free energy, which was negative in our study (Mao et al., 2023). VDWAALS<0 indicates that the hydrophobic action is conducive to binding, and EEL indicates that the polar solvation energy is negative; these results indicate that hydrogen bonding and other actions are conducive to binding. Total solvation-free energy (GSOLV) represents a positive value of total solvation-free energy, indicating that it is not conducive to binding. GSOLV represents the interaction between nonpolar solvation energy and polar solvation energy (EGB). EGB is a positive value, indicating that it does not combine well and that the nonpolar effect is beneficial to binding. MDS revealed that the binding between AKT1, IL-6, and TNF-α and quercetin is stable, further suggesting that quercetin plays an anti-atherosclerotic role *via* multiple targets.

In the present study, the main VO ingredients, potential pharmacological targets, and signal pathways were identified for treating AS using a computer-aided drug design approach. Our study results indicate that VO ameliorates multiple pathological features of AS *via* a direct synergistic effect on multiple targets and pathways. Further experiments are warranted to verify the biological mechanism by which VO regulates AS signaling pathways because there is still no evidence that VO antagonizes AS in animals or clinical experiments.

## 5 Conclusion

Using network pharmacology, molecular docking, and MDS, this study comprehensively and systematically revealed that the characteristics of the key ingredients in VO (including quercetin, luteolin, and kaempferol), potential targets (AKT1, IL-6, and TNF-α), various biological processes (response to xenobiotic stimulus, cellular response to lipid, and response to hormone), and multiple pathways (cancer, fluid shear stress and atherosclerosis, and lipid and atherosclerosis) prevent the occurrence and development of AS, providing an important theoretical basis for clinical treatment of AS and related research.

## Data availability statement

The datasets presented in this study can be found in online repositories. The names of the repository/repositories and accession number(s) can be found in the article/**Supplementary Material**.

## Author contributions

Data were processed by TYC, YYG, XJY, and XY. The manuscript was drafted by TYC and YYG. Data acquisition, analysis, and interpretation were conducted by WY. All authors listed have made a substantial, direct, and intellectual contribution to the work and approved it for publication.

## Supplementary material

Supplementary Figure 1 Molecular docking of VO main ingredients with AKT1, TNF-α and IL-6 in 2D binding posture. **(A)**: Two-dimensional (2D) binding posture schematic diagram of AKT1 and quercetin. **(B)**: 3D binding posture schematic diagram of AKT1 and luteolin. **(C)**: 2D binding posture schematic diagram of AKT1 and kaempferol. **(D)**: 2D binding posture schematic diagram of IL-6 and quercetin. **(E)**: 2D binding posture schematic diagram of IL-6 and luteolin. **(F)**: 2D binding posture schematic diagram of IL-6 and kaempferol. **(G)**: 2D binding posture schematic diagram of TNF-α and quercetin. **(H)**: 2D binding posture schematic diagram of TNF-α and luteolin. **(I)**: 2D binding posture schematic diagram of TNF-α and kaempferol. (TNF-α was selected for docking with TNF.)
