# Supplementary figures and images for: Key ingredients in Verbena officinalis and determination of their anti-atherosclerotic effect using a computer-aided drug design approach
Source: Front Plant Sci. 2023 Apr 3;14:1154266. doi: 10.3389/fpls.2023.1154266 (PMC10106644; doi:10.3389/fpls.2023.1154266)

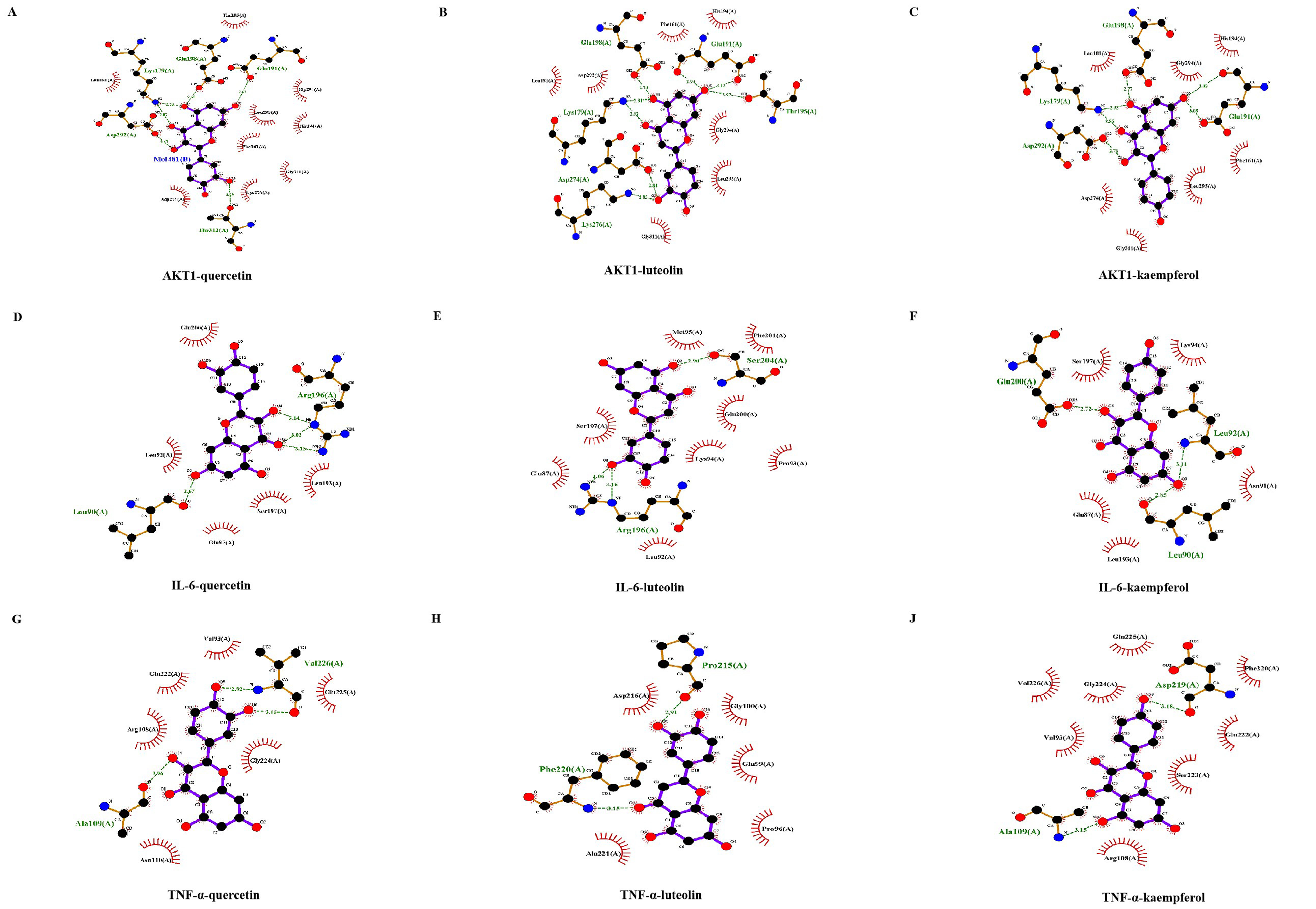

Supplement: Supplementary Figure 1 — Molecular docking of VO main ingredients with AKT1, TNF-α and IL-6 in 2D binding posture. (A): Two-dimensional (2D) binding posture schematic diagram of AKT1 and quercetin. (B): 3D binding posture schematic diagram of AKT1 and luteolin. (C): 2D binding posture schematic diagram of AKT1 and kaempferol. (D): 2D binding posture schematic diagram of IL-6 and quercetin. (E): 2D binding posture schematic diagram of IL-6 and luteolin. (F): 2D binding posture schematic diagram of IL-6 and kaempferol. (G): 2D binding posture schematic diagram of TNF-α and quercetin. (H): 2D binding posture schematic diagram of TNF-α and luteolin. (I): 2D binding posture schematic diagram of TNF-α and kaempferol. (TNF-α was selected for docking with TNF.) [file Image_1.jpeg]
