# Supplementary material for: Key ingredients in Verbena officinalis and determination of their anti-atherosclerotic effect using a computer-aided drug design approach
Source: Front Plant Sci. 2023 Apr 3;14:1154266. doi: 10.3389/fpls.2023.1154266 (PMC10106644; doi:10.3389/fpls.2023.1154266)
Supplement: Supplementary file 2 [file Table_1.docx]

https://www.jianguoyun.com/p/DcxHtFEQ9v6fCxiyvfIEIAA
